# Associated Factors of Hypertension in Women and Men in Vietnam: A Cross-Sectional Study

**DOI:** 10.3390/ijerph16234714

**Published:** 2019-11-26

**Authors:** Tran Quoc Cuong, Le Van Bao, Nguyen Anh Tuan, Vo Van Thang, Nguyen Minh Quan, Shwu-Huey Yang, Tuyen Van Duong

**Affiliations:** 1 Department of Anesthesiology, Thu Duc District Hospital, Ho Chi Minh City 713-11, Vietnam; quoccuong.mph@gmail.com; 2 School of Public Health, Military Medical University, Hanoi 121-08, Vietnam; ngabaott@gmail.com (L.V.B.); tuanytcc_hvqy@yahoo.com (N.A.T.); 3 Institute of Community Health Research, Faculty of Public Health, College of Medicine and Pharmacy, Hue University, Thua Thien Hue 491-20, Vietnam; vovanthang147@hueuni.edu.vn; 4 Director Office, Thu Duc District Hospital, Ho Chi Minh City 713-11, Vietnam; quan_minhnguyen@yahoo.com; 5 School of Nutrition and Health Sciences, Taipei Medical University, Taipei 110-31, Taiwan; sherry@tmu.edu.tw; 6 Research Center of Geriatric Nutrition, Taipei Medical University, Taipei 110-31, Taiwan; 7 Nutrition Research Center, Taipei Medical University Hospital, Taipei 110-31, Taiwan

**Keywords:** hypertension, added salts, older age, diabetes, overweight, obesity, abdominal obesity, smoking, poverty, Vietnam

## Abstract

Background: Hypertension is a direct cardiovascular disease risk. It causes a heavy burden on the healthcare system globally. We aim to assess hypertension occurrence and its associated factors among women and men in Vietnam. Methods: A cross-sectional study was conducted from January to February 2019 on 2203 community-dwelling women and men aged 18 years or above. Participants’ characteristics, comorbidity, behaviors, and physical measures were evaluated. Hypertension was classified as systolic/diastolic blood pressure ≥140/90 mmHg or using antihypertensive medication. We analyzed data using logistic regression models. Results: The prevalence of hypertension was 24.3% (20.9% in women, 29.1% in men). For women, older age (odds ratio, OR, 6.80–12.41; *p* < 0.001), income above the poverty line (OR, 0.64; *p* = 0.008), diabetes comorbid (OR, 2.98; *p* < 0.001), added salts consumption (OR, 1.80; *p* < 0.001), overweight/obesity (OR, 1.64; *p* = 0.005), abdominal obesity (OR, 2.07; *p* < 0.001) were associated with hypertension. For men, older age (OR, 2.67–5.92; *p* < 0.001), diabetes comorbid (OR, 2.25; *p* = 0.010), smoking (OR, 1.38; *p* = 0.046), and overweight/obesity (OR, 2.18; *p* < 0.001) were associated with hypertension. Conclusions: Hypertension is prevalent in Vietnamese people. The associated factors of hypertension are varied by gender.

## 1. Introduction

Non-communicable diseases (NCDs) place a heavy burden on the healthcare system across the globe [1]. It accounts for 71% of the total burden in Vietnam [2]. Among NCDs, cardiovascular disease (CVD) is a leading cause of premature death worldwide [1,2,3]. Hypertension is the strongest risk factor of CVD and causes a huge financial burden in Vietnam and globally [4,5,6,7]. Strategical interventions to reduce its burden could advance the sustainable development goals (SDGs), especially in low- and middle-income (LMIC) countries [8,9].

Globally, the prevalence of hypertension is high and striking from 28.5% in high-income countries to 31.5% in low- and middle-income countries [10]. Meanwhile, the awareness (46.5%–67.0%) and control (10.2%–37.9%) are inadequate in low-, middle-, and high-income countries [10,11,12]. In the Vietnamese population, blood pressure constantly increased from 2001–2009 [13]. The prevalence of hypertension in Vietnam was 18.4% based on three national surveys and 21.1% based on 10 other studies [14]. The level of hypertension awareness (9.3%–25.9%) and treatment (4.7%–12.2%) were very low [14,15]. In addition, the medication adherence rate (49.8%) was relatively low in hypertensive people [16].

The disease pattern in Vietnam has transited from communicable to non-communicable during the socioeconomic reforms [13,17,18]. Ho Chi Minh city is the most developed one in Vietnam with its fast socioeconomic change, which significantly contributed to this epidemiologic transition. Managing hypertension plays an important role in improving CVD outcomes and reducing its burden [4,5,6,19]. Therefore, it is important to identify the risk factors of hypertension, such as personal characteristics, dietary intake, physical activity, and lifestyle [20,21]. The previous studies in Vietnam were conducted a few years ago, which may not reflect the current status and determinant of hypertension [13,15]. In addition, these factors may influence hypertension differently in women and men, which are scarcely studied. We aim to assess hypertension occurrence and investigate the potential determinants of hypertension among women and men in Ho Chi Minh City, Vietnam.

## 2. Research Methods

### 2.1. Study Design and Settings

A cross-sectional study design was conducted between January and February 2019. The study participants were recruited from three communities named Hiep Binh Chanh, Linh Xuan, and Tam Phu communes in Thu Duc District, Ho Chi Minh City, Vietnam.

### 2.2. Sampling and Sample Size

The sample size was calculated using the formula (1) [22]:

(1) $$N=\frac{Z^{2}p\left(1-p\right)}{d^{2}}$$

where *N* is the sample size, *Z* is the level of confidence, *p* is expected prevalence, and *d* is the absolute error of precision, corresponding to effect size [22]. The sample of 384 was calculated with *Z* = 1.96 for type I error of 5%, *p* = 0.20 as the prevalence of hypertension was ranged from 18.4% to 21.1% in Vietnam [14], and *d* = 0.04 as suggested for a cross-sectional study design [23]. According to the World Health Organization guideline in the STEPwise approach to surveillance, in order to have an adequate precision level for each sex-age estimate, the sample size must be multiplied by 6 (3 groups of age: 18–44, 45–59, 60–69 years for men and women) [24]. The sample size was 384 × 6 = 2304. 

A convenience sampling method was utilized to recruit study participants in the community. People recruited in the current study were of the general public, who were aged 18 to 69 years, without any mental health issues, and able to read and understand the local language. People excluded were those with catastrophic diseases, such as chronic kidney disease, chronic obstructive pulmonary disease, cancer, cirrhosis, stroke, ischemic heart disease, or coronary artery disease. 

### 2.3. Measurements

#### 2.3.1. Participants’ Characteristics

Participants’ characteristics were studied, including age (18–44, 45–59, 60–69 years), gender (men vs. women), marital status (never married vs. ever married), education (elementary school and below, secondary school, high school and above), occupation (retirement, officers/workers/traders, and other), and income. The monthly income was classified into below the poverty line (<2.3 million VND/month), and above the poverty line (≥2.3 million VND/month), with 1 USD = 23,000 VND according to Vietcombank-State Bank of Vietnam [25,26]. In addition, diabetes and high cholesterol comorbidities were also investigated. 

#### 2.3.2. Health Behaviors

Participants were asked about their current health-related behaviors including tobacco smoking (yes vs. no); alcohol drinking (yes vs. no); fruits and vegetable intake (<5 servings/day vs. ≥5 servings/day), 1 serving is equal to 1 medium size piece of apple, banana, orange, or to ½ cup of chopped or cooked fruit, or to 1 cup of raw green leafy vegetables, or to ½ cup of other vegetables, cooked or chopped raw; added salt consumption (yes vs. no); and physical activity (yes vs. no).

#### 2.3.3. Blood Pressure

The parameters measured were systolic blood pressure (SBP) and diastolic blood pressure (DBP) in mmHg. The SBP and DBP were measured using a standard clinical manual aneroid sphygmomanometer (Yamasu Sphygmomanometer, Yamasu company, Tokyo, Japan). The blood pressure was measured three times with a 30-second interval on all participants after a 10 min rest, in the right arm, and in sitting position. The average value of three measurements was calculated. Participants were also asked whether or not they have been taking antihypertensive medication. Hypertension was classified as systolic blood pressure/diastolic blood pressure ≥140/90 mmHg [27,28,29], or using antihypertensive medication. The antihypertensive drugs commonly used by study participants were angiotensin-converting enzyme inhibitors (e.g., Captorile 25 mg), Ca-Antagonists (e.g., Amlor 5 mg), and β-blockers (Valsartan 80 mg).

#### 2.3.4. Anthropometrics

The anthropometric parameters including height (cm), weight (kg), waist circumference (cm), hip circumference (cm), were measured by doctors or nurses for all participants wearing light clothes and bare feet. Body weight was measured using a weighing scale (CSK-120, Nhon Hoa Scale Company, Ho Chi Minh, Viet Nam), height was reported by participants, waist circumference, and hip circumference were measured using a clinical tape measure. The parameters were recorded to nearest 0.1 kg, or 0.1 cm, appropriately. 

The body mass index (BMI) was calculated from weight (kg)/height (m)^2^. According to WHO classification for the Western Pacific region, BMI was classified as normal weight (<25.0 kg/m^2^), overweight, and obesity (≥25.0 kg/m^2^) [30,31]. In addition, abdominal obesity for South Asian people was defined as a waist –hip ratio ≥0.90 for men or ≥0.85 for women, respectively [32]. 

### 2.4. Data Collection Procedure

The interviewers (two medical doctors, and eight nurses) were well-trained about data collection by a senior researcher. A two-day training section was conducted in Thu Duc District Hospital. The permission was achieved from the District Health Center. Researchers then contacted local authority people and local volunteers to discuss the study. People satisfied with the recruitment criteria were invited to the survey. The face-to-face interviews were conducted in the participants’ houses using printed questionnaires. The blood pressure, height, weight, waist circumference, and hip circumference were measured by well-trained doctors and nurses. Each interview took about 15–30 min. There were 2203 people who agreed and voluntarily participated in the study and completed the assessment (response rate of 95.6%).

### 2.5. Ethical Consideration

The study was approved by the institutional review board of the National Institute of Hygiene and Epidemiology in Vietnam (NIHE-IRB-43/2018). All participants voluntarily participated and signed the inform consent form before their participation.

### 2.6. Data Analysis

The descriptive analysis was used to examine the distribution of hypertension, characteristics, behaviors, and physical parameters. The independent-samples t-test and Chi-square test were used to compare the distribution of hypertension among categories of studied variables, appropriately. Next, the bivariate logistic regression models were run to investigate the associated factors of hypertension. The Spearman correlation was used to check the correlation among predictors to avoid multicollinearity in multivariate regression models. Finally, the factors that showed the association with hypertension at *p* < 0.20 in the bivariate model were selected into the multivariate model [33]. The *p*-value < 0.05 was set as significance. We analyzed data using the IBM SPSS Version 20.0 (IBM Corp, Armonk, NY, USA) [34].

## 3. Results

The average age of the study population was 46.4 ± 13.5 years. The prevalence of hypertension was 24.3% (535 out of 2203 participants) for the total sample, 20.9% (268/1285) for women, and 29.1% (267/918) for men. Among the hypertensive participants, there were 279 (52.1%) using antihypertensive drugs. The proportion of hypertension significantly differed in different groups of age, gender, marital status, education, occupation, monthly income, comorbid diabetes, high cholesterol, smoking, drinking, added salts consumed, BMI, and abdominal obesity (Table 1). 

In the total sample, the odds of hypertension were significantly higher in people associated with the factors of older age, men, ever married, comorbid diabetes, high cholesterol, smoking, drinking, consuming added salts, and those with overweight and obesity. The odds of hypertension were significantly lower in people with higher education, a current job, and those above the poverty line (Table 2). To avoid the multicollinearity among the confounders, the correlation among the factors associated with hypertension at *p* < 0.20 in bivariate regression was examined. The results of the Spearman test showed that age was moderately correlated to marital status and occupation; gender was moderately correlated to smoking, drinking, and abdominal obesity; comorbid diabetes was moderately correlated to comorbid hypercholesterolemia (Table S1). Therefore, age, gender, education, income, comorbid diabetes, added salts, BMI, and abdominal obesity were included in the multivariate regression analysis. The result showed that, as compared with people aged 18–44 years, those aged 45–59 (odds ratio, OR, 3.99; 95% confidence interval, 95% CI, 3.01–5.30; *p* < 0.001), and aged 60–69 (OR, 8.37; 95% CI, 6.06–11.55; *p* < 0.001) had higher odds of hypertension. In comparison with women, men had higher odds of hypertension (OR, 2.32; 95% CI, 1.85–2.91; *p* < 0.001). The odds of hypertension were higher in people with comorbid diabetes (OR, 2.72; 95% CI, 1.85–4.00; *p* < 0.001). People consumed added salts had higher odds of hypertension (OR, 1.66; 95% CI, 1.33–2.07; *p* < 0.001) as compared with those who did not consume. Participants with BMI ≥ 25.0 kg/m^2^ had higher odds of hypertension (OR, 1.90; 95% CI, 1.48–2.44; *p* < 0.001), as compared with those with BMI < 25.0 kg/m^2^. People with abdominal obesity had higher odds of hypertension (OR, 1.71; 95% CI, 1.36–2.15; *p* < 0.001), as compared with normal people (Table 3).

In the women sample, the odds of hypertension were significantly higher in people associated with the factors of older age, ever married, comorbid diabetes, high cholesterol, consuming added salts, and those with overweight and obesity. The odds of hypertension were significantly lower in people with higher education, a current job, and those above the poverty line (Table 2). The results of the Spearman test showed that age was moderately correlated to education and occupation; comorbid diabetes was moderately correlated to high cholesterol (Table S1). Therefore, age, marital status, income, comorbid diabetes, added salts, BMI, and abdominal obesity were included in the multivariate regression analysis. The result showed that, as compared to women aged 18–44 years, those aged 45–59 (OR, 6.80; 95% CI, 4.02–11.49; *p* < 0.001), and aged 60–69 (OR, 12.41; 95% CI, 7.16–21.52; *p* < 0.001) had higher odds of hypertension. As compared to women with income at below poverty line level, those with income above the poverty line level had lower odds of hypertension (OR, 0.64; 95% CI, 0.46–0.89; *p* = 0.008). The odds of hypertension were higher in women with comorbid diabetes (OR, 2.98; 95% CI, 1.81–4.91; *p* < 0.001). Women who consumed added salts had higher odds of hypertension (OR, 1.80; 95% CI, 1.32–2.45; *p* < 0.001) as compared to those who did not consume. Women with BMI ≥ 25.0 kg/m^2^ had higher odds of hypertension (OR, 1.64; 95% CI, 1.16–2.33; *p* = 0.005), as compared with those with BMI < 25.0 kg/m^2^. Women with abdominal obesity had higher odds of hypertension (OR, 2.07; 95% CI, 1.49–2.87; *p* < 0.001), as compared to non-abdominal obesity women (Table 3).

In the men sample, the odds of hypertension were significantly higher in people associated with factors of older age, ever married, diabetes, hypercholesterolemia comorbid, smoking, and those with overweight and obesity. The odds of hypertension were significantly lower in people with higher education and a current job (Table 2). The results of the Spearman test showed that age was moderately correlated to marital status; comorbid diabetes was moderately correlated to high cholesterol (Table S1). Therefore, age, education, occupation, income, comorbid diabetes, smoking, BMI, and abdominal obesity were included in the multivariate regression analysis. The result showed that, as compared to men aged 18–44 years, those aged 45–59 (OR, 2.67; 95% CI, 1.85–3.87; *p* < 0.001) and aged 60–69 (OR, 5.92; 95% CI, 3.41–10.28; *p* < 0.001) had higher odds of hypertension. The odds of hypertension were higher in men with comorbid diabetes (OR, 2.25; 95% CI, 1.21–4.18; *p* = 0.010). Men who smoked had higher odds of hypertension than those who did not smoke (OR, 1.38; 95% CI, 1.01–1.90; *p* = 0.046). Men with BMI ≥ 25.0 kg/m^2^ had higher odds of hypertension (OR, 2.18; 95% CI, 1.52–3.13; *p* < 0.001), as compared to those with BMI < 25.0 kg/m^2^ (Table 3).

## 4. Discussion

In the total sample, men were 2.32 times more likely to have hypertension as compared to women. The prevalence of hypertension was 20.9% for women, and 29.1% for men. This was slightly higher than the finding from the pooled analysis from 200 countries, which showed that the prevalence of raised blood pressure was 20.1% for women and 24.1% for men [35]. In both women and men, those with older age were more likely to have hypertension. It was summarized that age and gender were the major risk factors of hypertension in Vietnam and other countries [15,36,37,38,39,40,41,42]. 

Women with income above the poverty line had less likelihood of having hypertension as compared to those with income below the poverty line. The association was not found in men. This was similar to the finding of a multilevel analysis in Colombia [43]. In addition, it was summarized that the association between socioeconomic status and hypertension was most consistent for women but less consistent for men [44]. The income/wealth-related inequity was reported as a key factor of hypertension screening, treatment, and control in many low-, middle-, and high-income countries [42,45,46,47]. The appropriate public health interventions are encouraged to narrow down the gap in economic and education that may be the strategical approach to improve public awareness and capacity of public health services in hypertension management. 

In both women and men, those with diabetes and hypercholesterolemia comorbidity were more likely to have hypertension. The coexistence of diabetes and hypertension was summarized [48]. Arterial stiffness indices (stiffness index and reflection index) were found to be a strong predictor of future CVD events [49]. In addition, in mothers with gestational diabetes mellitus and dyslipidemia, their child had a higher rate of obesity [50]. Therefore, the management of both blood glucose and blood pressure is a strategical way to prevent cardiovascular events or death [48,51]. The antihypertensive monotherapy with diuretics, Ca-antagonists, or zinc metabolism alterations showed the effect on lipid metabolism and inflammation, which were suggested in managing primary arterial hypertension [52]. The combination of pharmacology and zinc from dietary intake or supplements also showed an effect on hypertension management [53]. Moreover, the management of hypertension with different strategies was summarized as the primary prevention of cardiovascular diseases [54,55,56].

Lifestyle interventions were found as the primary prevention of hypertension and dyslipidemia, which further contributes to preventing cardiovascular diseases [54]. In the current study, smoking was analyzed among men, and the result showed a significantly positive association between smoking and hypertension. Smoking is a strong predictor of hypertension and cardiovascular diseases [57]. In addition, women as passive smokers have also been affected by smoke with a dose-response association [58]. Smoking cessation is a simple and effective lifestyle to prevent hypertension and cardiovascular events [57,59,60].

Salt intake was found as a key factor of hypertension in previous studies [37]. However, in the current study, the association was only found in women, but not in men. In a previous study in Vietnam, salt consumption was not significantly associated with high BP at the national level, but the association differed between urban and rural areas in Vietnam [61]. However, the evidence of this association was consistently found in other countries, namely in Brazil [62], China [63], in Cameroon [37]. Moreover, the long term effect of reduction in salt intake on population BP and CVD was summarized from several randomized trials [64,65,66].

Overweight and obesity classified by BMI showed a positive association with hypertension in both women and men. The abdominal obesity classified by WHR showed the possibility of hypertension in women. The associations of BMI and WHR with hypertension were found in previous studies [13,36,67,68,69]. In the current study, for men, the association of BMI and WHR with hypertension was found in bivariate analysis, but the association between WHR and hypertension was jeopardized in the multivariate analysis. The finding was supported by a previous study that all anthropometric indicators were associated with hypertension in women, but only BMI and its combination with other indicators showed a significant association in men [70]. 

The current study has some limitations. Firstly, the causality was not drawn as the cross-sectional nature of the design. Secondly, the sample was not a nation-wide one, and the generalizability was limited, even though the sample was adequate to explore the association and contribute to the evidence pool. In the present study, we only asked participants about their vegetables and fruits and salt consumption, which limited the investigation on the association between dietary intake and hypertension. In addition, the quantity of alcohol, salt consumption, and physical activity were not assessed, and the pulse rate was not recorded in the current study. Future studies are suggested to conduct in a larger population and with a longitudinal design in order to have a comprehensive assessment and examine the causal relationship between potential factors and hypertension. 

## 5. Conclusions

Hypertension was prevalent in the study population. The associated factors of hypertension were age, gender, income, diabetes comorbidity, smoking, salt consumption, and obesity. The associations were varied by gender. The associated factors of hypertension in women and men were studied, which provided important evidence for effective screenings and potential interventions for the treatment and control of hypertension.

## Figures and Tables

**Table 1:** Participants’ characteristics, history of diseases, behaviors, and physical parameters.

Variables	Total (*N* = 2203)	Non-HTN (*N* = 1668)	HTN (*N* = 535)	*p*-Value *
Age groups				<0.001
18–44	957 (43.4)	867 (52.0)	90 (16.8)	
45–59	820 (37.3)	572 (34.3)	248 (46.4)	
60–69	426 (19.3)	229 (13.7)	197 (36.8)	
Gender				<0.001
Women	1285 (58.3)	1017 (61.0)	268 (50.1)	
Men	918 (41.7)	651 (39.0)	267 (49.9)	
Marital status				<0.001
Never married	313 (14.2)	283 (17.0)	30 (5.6)	
Ever married	1890 (85.8)	1385 (83.0)	505 (94.4)	
Education				<0.001
Elementary school or below	368 (16.7)	263 (15.8)	105 (19.6)	
Secondary school	675 (30.6)	483 (29.0)	192 (35.9)	
High school and above	1160 (52.7)	922 (55.2)	238 (44.5)	
Occupation				<0.001
Retirement	421 (19.1)	240 (14.4)	181 (33.8)	
Officers/Workers/Traders	910 (41.3)	776 (46.5)	134 (25.0)	
Others	872 (39.6)	652 (39.1)	220 (41.1)	
Monthly income				<0.001
Below poverty line	488 (22.2)	337 (20.2)	151 (28.2)	
Above poverty line	1715 (77.8)	1331 (79.8)	384 (71.8)	
Comorbidities				
Diabetes mellitus				<0.001
No	2064 (93.7)	1611 (96.6)	453 (84.7)	
Yes	139 (6.3)	57 (3.4)	82 (15.3)	
Hypercholesterolemia				<0.001
No	1920 (87.2)	1529 (91.7)	391 (73.1)	
Yes	283 (12.8)	139 (8.3)	144 (26.9)	
Health behaviors				
Smoking tobacco				<0.001
No	1805 (81.9)	1404 (84.2)	401 (75.0)	
Yes	398 (18.1)	264 (15.8)	134 (25.0)	
Drinking alcohol				0.003
No	1694 (76.9)	1308 (78.4)	386 (72.1)	
Yes	509 (23.1)	360 (21.6)	149 (27.9)	
Consuming added salts				0.046
No	1095 (49.7)	858 (51.4)	237 (44.3)	
Yes	1108 (50.3)	810 (48.6)	298 (55.7)	
Daily intake of fruits and vegetables ^†^				0.831
<5 servings/day	1937 (87.9)	1468 (88.0)	469 (87.7)	
≥5 servings/day	266 (12.1)	200 (12.0)	66 (12.3)	
Exercise				0.246
No	883 (40.1)	680 (40.8)	203 (37.9)	
Yes	1320 (59.9)	988 (59.2)	332 (62.1)	
Anthropometrics				
Height, cm, mean ± SD	159.5 ± 7.2	159.4 ± 7.1	159.9 ± 7.4	0.229
Weight, kg, mean ± SD	58.5 ± 14.2	57.3 ± 9.0	62.2 ± 23.7	<0.001
BMI, kg/m^2^, mean ± SD	22.9 ± 5.1	22.5 ± 2.9	24.3 ± 9.0	<0.001
BMI groups				<0.001
Normal (<25.0 kg/m^2^)	1758 (79.5)	1390 (83.3)	368 (68.8)	
Overweight/obesity (≥25.0 kg/m^2^)	445 (20.2)	278 (16.7)	167 (31.2)	
WC, cm, mean ± SD	78.5 ± 10.9	76.6 ± 10.2	84.4 ± 10.7	<0.001
HC, cm, mean ± SD	90.3 ± 8.7	89.4 ± 8.7	93.0 ± 8.4	<0.001
WHR, %, mean ± SD	87.0 ± 9.3	85.8 ± 9.3	90.7 ± 8.1	<0.001
Abdominal Obesity ^‡^				<0.001
Normal	1114 (50.6)	917 (55.0)	197 (36.8)	
Abdominal obesity	1089 (49.4)	751 (45.0)	338 (63.2)	
SBP, mmHg, mean ± SD	123.3 ± 15.8	117.6 ± 10.3	140.9 ± 17.0	<0.001
DBP, mmHg, mean ± SD	77.2 ± 10.1	74.2 ± 7.5	86.7 ± 11.0	<0.001

Abbreviations: HTN, hypertension; BMI, body mass index; WC, waist circumference; HC, hip circumference; WHR, waist–hip ratio; SBP, systolic blood pressure; DBP, diastolic blood pressure. * Data were presented as frequency and percentage, mean ± SD, and *p*-values were calculated using Chi-square test, and independent sample *t*-test, appropriately. ^†^ 1 serving is equal to 1 medium size piece of apple, banana, orange, or to ½ cup of chopped or cooked fruit, or to 1 cup of raw green leafy vegetables, or to ½ cup of other vegetables, cooked or chopped raw. ^‡^ Abdominal obesity was defined as a waist–hip ratio ≥0.90 for men, ≥0.85 for women.

**Table 2:** The associated factors of hypertension via simple logistic regression models.

Variables	Overall (*N* = 2203)		Women (*N* = 1285)		Men (*N* = 918)	
	OR (95% CI)	*p*-Value	OR (95% CI)	*p*-Value	OR (95% CI)	*p*-Value
Age groups						
18–44	Reference		Reference		Reference	
45–59	4.18 (3.21–5.44)	<0.001	8.88 (5.39–14.63)	<0.001	3.21 (2.28–4.51)	<0.001
60–69	8.29 (6.21–11.06)	<0.001	16.89 (10.08–28.28)	<0.001	7.75 (5.09–11.81)	<0.001
Gender						
Women	Reference					
Men	1.56 (1.28–1.89)	<0.001				
Marital status						
Never married	Reference		Reference		Reference	
Ever married	3.44 (2.33–5.08)	<0.001	3.18 (1.73–5.84)	<0.001	4.18 (2.50–6.97)	<0.001
Education						
Elementary school or below	Reference		Reference		Reference	
Secondary school	1.00 (0.75–1.32)	0.976	0.94 (0.66–1.34)	0.743	0.95 (0.59–1.54)	0.848
High school and above	0.65 (0.50–0.85)	0.001	0.54 (0.38–0.77)	0.001	0.63 (0.40–0.99)	0.043
Occupation						
Retirement	Reference		Reference		Reference	
Officers/Workers/Traders	0.23 (0.18–0.30)	<0.001	0.20 (0.14–0.28)	<0.001	0.21 (0.14–0.32)	<0.001
Others	0.45 (0.35–0.57)	<0.001	0.46 (0.34–0.64)	<0.001	0.34 (0.23–0.52)	<0.001
Monthly income						
Below poverty line	Reference		Reference		Reference	
Above poverty line	0.64 (0.52–0.81)	<0.001	0.52 (0.39–0.69)	<0.001	0.79 (0.55–1.13)	0.195
Comorbidities						
Diabetes						
No	Reference		Reference		Reference	
Yes	5.12 (3.59–7.29)	<0.001	6.28 (3.97–9.92)	<0.001	4.03 (2.30–7.06)	<0.001
Hypercholesterolemia						
No	Reference		Reference		Reference	
Yes	4.05 (3.13–5.24)	<0.001	5.18 (3.67–7.32)	<0.001	3.03 (2.04–4.48)	<0.001
Health behaviors						
Smoking tobacco						
No	Reference				Reference	
Yes	1.78 (1.40–2.25)	<0.001			1.52 (1.14–2.03)	0.004
Drinking alcohol						
No	Reference				Reference	
Yes	1.40 (1.12–1.75)	0.003			1.20 (0.90–1.60)	0.208
Consuming added salts						
No	Reference		Reference		Reference	
Yes	1.33 (1.10–1.62)	0.004	1.54 (1.17–2.02)	0.002	1.18 (0.89–1.57)	0.253
Daily intake of fruits and vegetables *						
<5 servings/day	Reference		Reference		Reference	
≥5 servings/day	1.03 (0.77–1.39)	0.831	0.80 (0.51–1.26)	0.336	1.26 (0.84–1.89)	0.272
Exercise						
No	Reference		Reference		Reference	
Yes	1.13 (0.92–1.38)	0.246	1.10 (0.84–1.44)	0.507	1.07 (0.79–1.45)	0.654
Anthropometrics						
BMI groups						
Normal (<25.0 kg/m^2^)	Reference		Reference		Reference	
Overweight/obesity (≥25.0 kg/m^2^)	2.269 (1.814–2.838)	<0.001	2.37 (1.73–3.24)	<0.001	2.08 (1.50–2.86)	<0.001
Abdominal Obesity ^†^						
Normal	Reference		Reference		Reference	
Abdominal obesity	2.10 (1.71–2.56)	<0.001	2.53 (1.89–3.38)	<0.001	1.92 (1.44–2.56)	<0.001

Abbreviations: OR, odds ratio; CI, confidence interval; BMI, body mass index. * 1 serving is equal to 1 medium size piece of apple, banana, orange, or to ½ cup of chopped or cooked fruit, or to 1 cup of raw green leafy vegetables, or to ½ cup of other vegetables, cooked or chopped raw. ^†^ Abdominal obesity was defined as a waist–hip ratio ≥ 0.90 for men, ≥ 0.85 for women.

**Table 3:** The associated factors of hypertension via multivariate logistic regression models.

Variables	Overall (*N* = 2203)		Women (*N* = 1285)		Men (*N* = 918)	
	OR (95% CI)	*p*-Value	OR (95% CI)	*p*-Value	OR (95% CI)	*p*-Value
Age groups						
18–44	Reference		Reference		Reference	
45–59	3.99 (3.01–5.30)	<0.001	6.80 (4.02–11.49)	<0.001	2.67 (1.85–3.87)	<0.001
60–69	8.37 (6.06–11.55)	<0.001	12.41 (7.16–21.52)	<0.001	5.92 (3.41–10.28)	<0.001
Gender						
Women	Reference					
Men	2.32 (1.85–2.91)	<0.001				
Marital status						
Never married			Reference			
Ever married			1.05 (0.52–2.12)	0.893		
Education						
Elementary school or below	Reference				Reference	
Secondary school	1.26 (0.92–1.72)	0.147			1.09 (0.65–1.84)	0.734
High school and above	1.06 (0.78–1.44)	0.700			0.92 (0.55–1.52)	0.738
Occupation						
Retirement					Reference	
Officers/Workers/Traders					0.69 (0.39–1.21)	0.191
Others					0.88 (0.52–1.48)	0.620
Monthly income						
Below poverty line	Reference		Reference		Reference	
Above poverty line	0.80 (0.62–1.03)	0.080	0.64 (0.46–0.89)	0.008	1.17 (0.77–1.77)	0.459
Diabetes comorbidity						
No	Reference		Reference		Reference	
Yes	2.72 (1.85–4.00)	<0.001	2.98 (1.81–4.91)	<0.001	2.25 (1.21–4.18)	0.010
Health behaviors						
Smoking tobacco						
No					Reference	
Yes					1.38 (1.01–1.90)	0.046
Consuming added salts						
No	Reference		Reference			
Yes	1.66 (1.33–2.07)	<0.001	1.80 (1.32–2.45)	<0.001		
Anthropometrics						
BMI groups						
Normal (<25.0 kg/m^2^)	Reference		Reference		Reference	
Overweight/obesity (≥25.0 kg/m^2^)	1.90 (1.48–2.44)	<0.001	1.64 (1.16–2.33)	0.005	2.18 (1.52–3.13)	<0.001
Abdominal Obesity *						
Normal	Reference		Reference		Reference	
Abdominal obesity	1.71 (1.36–2.15)	<0.001	2.07 (1.49–2.87)	<0.001	1.27 (0.92–1.76)	0.142

Abbreviations: BMI, body mass index. * Abdominal obesity was defined as a waist–hip ratio ≥ 0.90 for men, ≥ 0.85 for women.

## Supplementary Materials

The following are available online at https://www.mdpi.com/1660-4601/16/23/4714/s1, Table S1: The Spearman correlation among the covariates.
