# Supplementary material for: Associated Factors of Hypertension in Women and Men in Vietnam: A Cross-Sectional Study
Source: Int J Environ Res Public Health. 2019 Nov 26;16(23):4714. doi: 10.3390/ijerph16234714 (PMC6926662; doi:10.3390/ijerph16234714)
Supplement: Supplementary file 1 [file ijerph-16-04714-s001.pdf]

Table S1. The Spearman correlation among the covariates.

	Age	Gender	Marital status	Education	Occupation	Income	Diabetes	High TC	Smoking	Drinking	Added salts	BMI
**Overall (*N*=2203)**												
Gender	-.129											
Marital status	**.335**	-.102										
Education	-.281	.149	-.206									
Occupation	**-.311**	.082	-.142	-.022								
Income	-.168	.070	.052	.185	-.042							
Diabetes	.208	-.011	.052	-.048	-.114	.004						
High TC	.208	.003	.117	-.027	-.151	.006	**.347**					
Smoking	-.002	**.505**	.059	-.017	.065	.026	-.010	.007				
Drinking	-.109	**.531**	-.055	.126	.096	.090	-.054	.002	**.431**			
Added salts	-.028	-.033	.001	-.013	.042	-.030	-.003	.007	-.015	.015		
BMI	.066	.056	.062	-.054	-.041	-.037	.079	.182	.008	.011	-.011	
Abdominal obesity	.174	-.079	.155	-.058	-.036	-.004	.136	.177	-.014	-.040	-.112	.181
**Women (*N*=1285)**												
Marital status	.285											
Education	**-.313**		-.233									
Occupation	**-.350**		-.125	.058								
Income	-.169		.021	.211	-.025							
Diabetes	.219		.044	-.099	-.131	-.003						
High TC	.236		.092	-.061	-.164	-.025	**.352**					
Added salts	-.007		.012	-.002	.045	-.035	.048	.029				
BMI	.130		.091	-.092	-.075	-.071	.087	.163			-.010	
Abdominal obesity	.148		.133	-.030	.009	-.005	.143	.166			-.110	.169
**Men (*N*=918)**												
Marital status	**.380**											
Education	-.201		-.147									
Occupation	-.235		-.147	-.174								
Income	-.147		.110	.118	-.085							
Diabetes	.197		.061	.037	-.085	.015						
High TC	.175		.150	.026	-.138	.052	**.340**					
Smoking	.106		.185	-.178	.055	-.013	-.024	.001				
BMI	-.002		.045	-.025	-.010	.002	.070	.207	-.038			
Abdominal obesity	.187		.168	-.075	-.089	.011	.123	.194	.045			.209

Abbreviations: TC, total cholesterol; CVD, cardiovascular disease; BMI, body mass index.
